# Efficacy and Safety of Upadacitinib in Rheumatoid Arthritis: Real-Life Experience from a Prospective Longitudinal Multicentric Study

**DOI:** 10.3390/jcm13020401

**Published:** 2024-01-11

**Authors:** Caterina Baldi, Simone Parisi, Paolo Falsetti, Jurgen Sota, Maria Chiara Ditto, Marco Capassoni, Miriana D’alessandro, Edoardo Conticini, Francesca Nacci, Clara Lisa Peroni, Laura Cometi, Enrico Fusaro, Bruno Frediani, Serena Guiducci

**Affiliations:** 1 Rheumatology Unit, Department of Medicine, Surgery and Neurosciences, University of Siena, 53100 Siena, Italy; catebaldi3@gmail.com (C.B.); paolo.falsetti@virgilio.it (P.F.); sota@student.unisi.it (J.S.); conticini.edoardo@gmail.com (E.C.); fredianibruno60@gmail.com (B.F.); 2 Rheumatology Unit, Azienda Ospedaliera Universitaria Città Della Salute e Della Scienza di Torino, 10126 Torino, Italy; mariachiaraditto@gmail.com (M.C.D.); cperoni@cittadellasalute.to.it (C.L.P.); fusaro.reumatorino@gmail.com (E.F.); 3 Rheumatology Unit, Department of Experimental and Clinical Medicine, University of Florence, 50121 Firenze, Italy; marco.capassoni@gmail.com (M.C.); dott.nacci@icloud.com (F.N.); lauracometi@gmail.com (L.C.); serena.guiducci@unifi.it (S.G.); 4 Respiratory Disease and Lung Transplantation Unit, University of Siena, 53100 Siena, Italy; dalessandro.miriana@gmail.com

**Keywords:** rheumatoid arthritis, tsDMARD, upadacitinib, JAKi, ultrasound

## Abstract

Background: We provide the first prospective longitudinal multicenter experience on Upadacitinib efficacy and safety profile in Rheumatoid Arthritis (RA) in a real-life context, focusing on clinimetric and ultrasonographic (US) data. Methods: RA patients referred to three Italian tertiary Centers who started Upadacitinib were enrolled as per ACR/EULAR classification criteria and prospectively reviewed. The primary aim of this study was to assess changes in clinimetric and ultrasonographic scores through time (at baseline, after 1 month, 3 months, and 6 months from the beginning of the therapy). Secondary aims were to: (i) estimate the impact of biologic lines of treatment and concomitant therapies on response to therapy; (ii) explore changes in laboratory parameters; and (iii) find potential predictive factors associated with response to therapy. Results: Seventy-one patients (49 Females and 22 Males) were included. Clinimetric scores, including the Disease Activity Score (DAS28-CRP) and Simplified Clinical Disease Activity Index (SDAI), and US findings (synovial hypertrophy and power Doppler) significantly improved (*p* = 0.029, *p* = 0.001, *p* = 0.001, *p* = 0.001, respectively). Regression analysis revealed a significant association between the concomitant csDMARDs therapy at baseline and the lack of improvement in synovial hypertrophy [OR −4.824, *p* = 0.010] as well as with DAS28-CRP [OR −0.690, *p* = 0.045], whereas the presence of increased ESR or CRP at baseline was able to predict a significant improvement in SDAI [OR 8.481, *p* = 0.003]. No adverse events, such as deep venous thrombosis, pulmonary embolism, or herpes zoster virus infection, were reported during this study observation. Conclusion: Our real-life experience confirms the efficacy of Upadacitinib in terms of clinical and ultrasonographic improvement, as well as displaying a good safety profile.

## 1. Introduction

Rheumatoid arthritis (RA) is a chronic autoimmune disease characterized by progressive joint damage as well as extra-articular features responsible for several comorbidities [1]. Its prevalence in Europe and North America is between 0.5% and 1.0%, affecting predominantly women, with a peak incidence around 50 years of age. Debates continue about the changing incidence of RA, with some reports indicating a decline in the second half of the 20th century and a possible increase after 1995. The reasons for these changes, possibly environmental ones, remain unclear. RAs concept has evolved over centuries, initially including conditions like gout and spondyloarthritis until the mid-20th century. Since then, RAs classification criteria and typical presentation have slightly changed [2].

Therefore, it carries severe effects on the overall quality of life and leads to a high socio-economic burden. Safe and effective long-term treatments are needed to reduce disease symptoms, prevent irreversible joint damage, and reduce the burden of the disease related to its comorbidities [1,3].

Conventional disease-modifying anti-rheumatic drugs (csDMARDs), such as methotrexate (MTX), represent the cornerstones of treatment. The expanding therapeutic choices have witnessed important breakthroughs with the development of biotechnologic drugs inhibiting the costimulatory signal or blocking pivotal cytokines like tumor necrosis factor (TNF)-α and interleukin-6. For instance, combination therapy with MTX and anti-TNF agents has enabled disease control in many patients unresponsive to csDMARDs alone [4,5]. Despite a considerable outcome improvement in the last two decades, more than half of the patients fail to achieve remission or low disease activity, and many patients experience safety and tolerability issues requiring treatment suspension [6,7,8,9,10,11].

Therefore, additional treatments have been developed to overcome this unmet need. In this regard, the members of the Janus kinasi (JAK) family represent intriguing targets for the treatment of RA patients that might prevent irreversible joint damage while reducing disease burden [12]. Currently, a host of trials have explored the inhibition of JAK for the treatment of RA. Upadacitinib (UPA), a JAK inhibitor engineered for greater selectivity towards JAK1, has demonstrated a favorable benefit-to-risk profile in patients with an inadequate response to csDMARDs and biologic agents [13,14,15,16]. In RA, the efficacy and safety of Upadacitinib were studied in patients with moderately to severely active disease, as the efficacy of Upadacitinib has been reported from five randomized controlled trials (RCTs), among which the phase III SELECT clinical program with approximately 4400 patients. The SELECT program evaluated the efficacy and safety of upadacitinib 15 mg once daily (which is, to date, the approved dose for RA in the USA and the EU) as monotherapy and in combination with csDMARDs, compared with placebo, methotrexate, and adalimumab, based on pooled data from five pivotal upadacitinib phase III clinical trials (SELECT-BEYOND [16], SELECT-COMPARE [17,18], SELECT-NEXT [19], SELECT-MONOTHERAPY [20], and SELECT-EARLY [21]. Two additional studies were included in the SELECT program: SELECT-CHOICE [22,23], which evaluated the safety and efficacy of upadacitinib versus abatacept in bDMARD-IR patients, and SELECT-SUNRISE [24], which evaluated upadacitinib in Japanese patients.

Until now, real-life data on the efficacy and safety of Upadacitinib in rheumatoid arthritis (RA) patients have been scarce. This study presents the first prospective multicenter experience evaluating Upadacitinib’s efficacy and safety in RA within a real-life setting, focusing on clinical and imaging (ultrasound) data. The aim of this study was to assess the response to Upadacitinib therapy by examining changes in disease activity indices (DAS28-CRP and SDAI) and ultrasound parameters (GS and PD) during the follow-up period compared to the baseline.

## 2. Materials and Methods

### 2.1. Study Design and Participants

Patients with rheumatoid arthritis (RA) who were scheduled to begin treatment with Upadacitinib at a dose of 15 mg/day were sequentially enrolled from three major Italian tertiary centers in this longitudinal observational study.

Inclusion criteria:

- Patients aged over 18 years
- Patients were classified as having RA according to the 2010 ACR/EULAR classification criteria [25].
- Inadequate responders, as for EULAR response criteria [26], require at least 6 months of treatment with MTX at the standard dosage (patients bio-naïve) or with MTX and bDMARDs at the standard dosage (patients bio-failure).

Exclusion criteria:

- The treatment with any bDMARDs and tsDMARDs other than upadacitinib.
- Allergy or intolerance to Upadacitinib

The following demographic, clinical, and therapeutic data were collected at baseline (BL, at the prescription of Upadacitinib) and after 1, 3, and 6 months of follow-up (1M, 3M, 6M, respectively): age, gender, body mass index (BMI), disease duration, concomitant treatments, the presence of comorbidities (hypertension, dyslipidemia, positive history for cardiovascular events), use of oral contraceptives, serum biomarkers including rheumatoid factor and anti-cyclic citrullinated peptide, physician’s global assessment of disease activity (PhGA), patient’s assessment of disease activity (PtGA), patient’s assessment of pain (VAS pain), health assessment questionnaire (HAQ), morning stiffness, Disease Activity Score (DAS28-CRP) and Simplified Clinical Disease Activity Index (SDAI) [27,28].

Ultrasound examination was carried out by four rheumatologists (C.B., P.F., M.C., and S.P.) with several years of experience (between 5 and 20 years) in musculoskeletal US, blinded to clinical conditions, and a good to excellent reliability (weighted kappa ≥ 0.7) was required before contributing to research studies. US was performed using an Esaote (Genoa, Italy) MyLab X8 eXP machine equipped with linear 4–15 and 8–24 MHz transducers.

Standardized B-mode and Power Doppler (PD) settings were optimized for all examinations (factory preset of the machines for musculoskeletal or small parts). Doppler parameters were: pulse repetition frequency within 500–750 Hz; Doppler frequency adapted to depth (generally within 7–11.1 MHz); and a color gain just under the artifacts limit.

All the patients were evaluated with a routine multi-site (20 joint sites) bilateral examination of hands (proximal interphalangeal joints (PIPs) 1–5), metacarpophalangeal joints (MCPs) 2–5, wrists, elbows, shoulders (gleno-humeral), hips, knees, ankles (tibiotalar), and feet (metatarsophalangeal joints (MTPs) 1–5). All joints were assessed following an internationally approved scanning protocol at baseline and at months 1, 3, and 6 [29,30,31].

For each joint site, the presence of gray scale (GS) and synovial vascularization (assessed using PD) were scored according to the 0–3 semiquantitative OMERACT-EULAR-PDUS scale [29].

Moreover, the presence of tenosynovitis of the extensor ulnaris carpi, flexor radialis carpi, extensor digitorum communis tendon, and flexor digitorum tendons was scored using the 0–3 semiquantitative scale.

The sum of all scores (GS, PD, and tenosynovitis) at all the joint sites for each patient was recorded for statistical purposes.

All patients were systematically followed up every 1–3 and 6 months and additionally during disease flares and/or safety issues. Before starting treatment with Upadacitinib, patients were screened to rule out active or latent infections by undergoing a complete medical examination, chest X-ray film, interferon gamma release assay, evaluation of hepatitis B and hepatitis C virus markers, and urine culture.

Upadacitinib was administered at a daily dosage of 15 mg.

Enrolled patients were on a stable dose of low- to medium-dose glucocorticoids (GC) (<10 mg/day prednisone or equivalent) and on-demand nonsteroidal anti-inflammatory drugs throughout this study period.

### 2.2. Aims and Endpoints

The main aim of this study was to assess the efficacy of Upadacitinib in treating RA by monitoring changes in disease activity indices (DAS28-CRP and SDAI) and ultrasound measurements (GS and PD), comparing these parameters from baseline through the follow-up period.

The secondary objectives were to evaluate:

1. Confounding variables as predictors of response to Upadacitinib therapy (line of therapy, concomitant csDMARDs therapy, RF and ACPA seropositivity, concomitant glucocorticoid therapy);
2. A safety profile (Drug adverse events (AEs)) was recorded to describe the safety profile.

### 2.3. Statistical Analysis

Data were analyzed using IBM-SPSS Statistics for Windows, version 28 (IBM Corp., Armonk, NY, USA). Descriptive statistics were employed to display the mean and standard deviation (SD) or median and interquartile range (IQR) as appropriate. The Shapiro-Wilk test was used to assess the normality of our data. Differences between groups for repeated variables were investigated by the Friedman test, followed by post-hoc analysis with the Wilcoxon Sign test. Linear logistic regression analysis was employed to predict the response to treatment. The threshold for statistical significance was set to *p* < 0.05, and all *p*-values were two-sided.

## 3. Results

Seventy-one patients (49 females and 22 males) affected by RA and treated with UPA were consecutively enrolled between January 2021 and July 2022. Demographic and clinical data are summarized in Table 1. The median ± IQR age of our cohort was 58.00 ± 10.50 years, and the median disease duration was 7.50 years (IQR ± 9.67 years). Forty subjects were treated with concomitant csDMARDs at the start of this study. The remaining 31 patients were not assigned csDMARD therapy due to adverse events (25 due to intolerance and 6 due to ineffectiveness) and by physician choice. The mean ± SD dose of methotrexate in patients receiving the combination therapy was 12.81 ± 3.12 mg every week. Fifteen patients were naïve to treatment with biologic agents or small molecules, whereas 56 patients had been previously treated with other biologic agents. Ten patients received Upadacitinib as a second-line therapy, 10 as a third-line therapy, 18 as a fourth-line therapy, 9 as a fifth-line treatment, 8 as a sixth-line treatment, and 1 as an eighth-line treatment. The number of patients continuing and discontinuing treatment with Upadacitinib according to sex, antibody profile, and HZV infection is summarized in Table 2.

**Figure 1:**
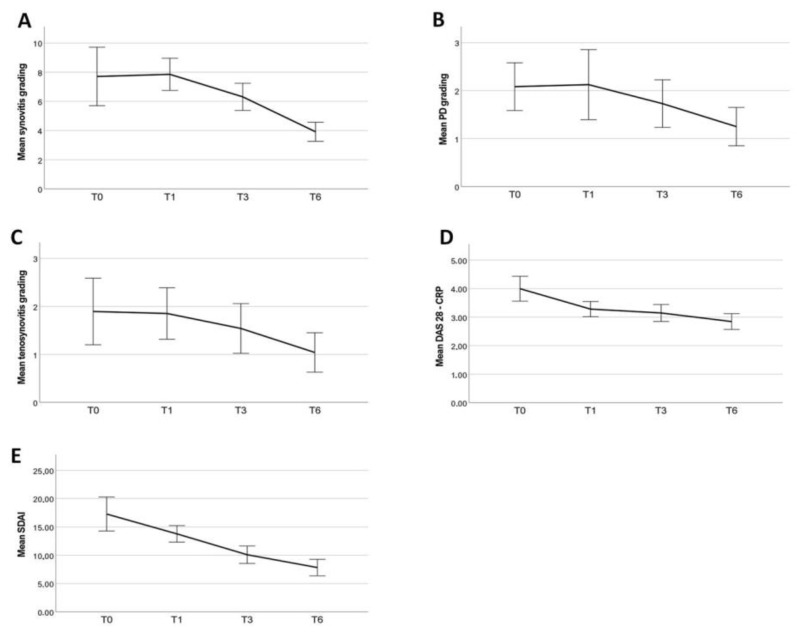
Ultrasonographic and clinimetric changes throughout this study period: Synovitis grading (**A**), power Doppler (PD) grading (**B**), tenosynovitis grading (**C**), Disease activity score (DAS) 28-CRP (**D**), and Simplified clinical disease activity index (SDAI) (**E**).

DAS28-CRP and SDAI, and US findings such as GS, PD, and tenosynovitis grade significantly improved throughout this study period (Table 3, Figure 1). HAQ, VAS pain, PtGA, PhGA, and morning stiffness also improved between baseline and the last follow-up assessment (*p* = 0.029, *p* < 0.001, *p* < 0.001, *p* < 0.001, *p* < 0.001, respectively).

Biologic-naive patients were significantly associated with a better improvement in SDAI score compared to patients already exposed to previous biologic agents (*p* = 0.018). Patients treated with Upadacitinib in monotherapy displayed a significantly higher improvement in GS grading (*p* = 0.019) and SDAI score (*p* = 0.004) compared to the patients treated with UPA and csDMARDs.

**Figure 2:**
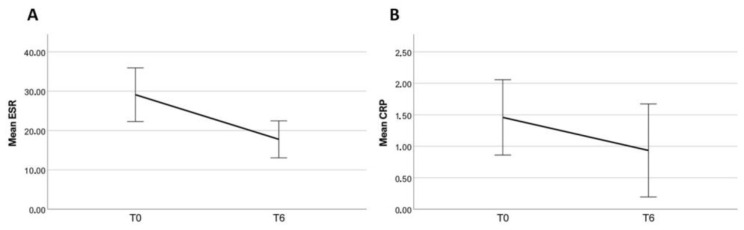
Impact of Upadacitinib on inflammatory markers: erythrocyte sedimentation rate (ESR) (**A**), C-reactive protein (CRP) (**B**). Mann–Witney test for medians.

Inflammatory markers including ESR and CRP significantly decreased from baseline to the last follow-up assessment (*p* < 0.001 and *p* = 0.001, respectively, Figure 2).

Regression analysis revealed a significant association between the concomitant csDMARDs therapy at baseline and the lack of improvement in grey scale (GS) [OR −4.824 C.I. (−8.427 −1.221), *p* = 0.010] and DAS28-CRP [OR −0.690 C.I. (−1.346–0.014), *p* = 0.045], whereas the presence of increased ESR or CRP at baseline was able to predict a significant improvement in SDAI [OR 8.481 C.I. (2.940–14.022), *p* = 0.003]. Table 4 provides detailed information for each variable used in the regression model for both US scores and clinimetric indexes.

**Figure 3:**
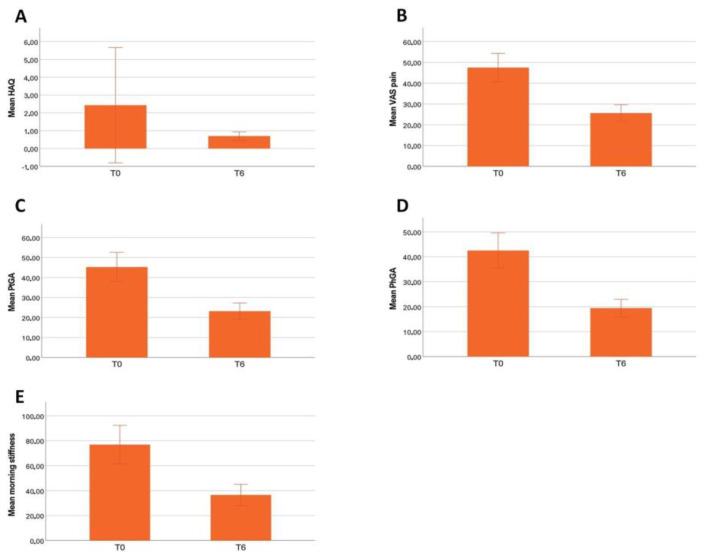
Improvement from baseline to last follow-up assessment of health assessment questionnaire (HAQ) (**A**), visual analogue scale (VAS) (**B**), patient global assessment (PtGA) (**C**), physician global assessment (PhGA) (**D**), and morning stiffness (**E**). HAQ, VAS pain, PtGa, and Morning stiffness were calculated and compared using the Wilcoxon signed rank test.

A significant improvement in all the activity indexes and clinimetric scores was observed already after the first month of Upadacitinib treatment and continued throughout the 3M and 6M follow-up visits. (Table 3, Figure 1, Figure 2 and Figure 3). The percentage of patients having remission according to a SDAI (simplified disease activity index) of less than 3.3 was: 3% (2/71) patients at one month (1M), 13% (9/71) at 3M, and 19% (12/64) at 6M. SDAI low disease activity (LDA < 3.3–11) was registered in 17 (24%), 35 (49%), and 38 (59%) patients at the respective timepoints. Prednisone daily intake did not significantly change during this study period (*p* = 0.551). Median GC doses (± IQR) at baseline, 3 months, and 6 months follow-up were equal to 0.00 (±5.00 mg), 0.63 (±5.00 mg), and 0.00 (±5.00 mg), respectively. Approximately 9 patients out of 29 discontinued steroid therapy at 6 months.

Seven patients discontinued therapy; among them, two interrupted the treatment due to adverse events (increase in levels of low-density lipoprotein (LDL) cholesterol and high-density lipoprotein (HDL) cholesterol; *Pseudomonas aeruginosa* urinary tract infection), while three subjects had to discontinue the therapy due to inefficacy; and lastly, the remaining two patients interrupted the administrations for non-medical reasons. No adverse events, including Varicella Zoster Virus (VZV) reactivation, deep venous thrombosis (DVT), pulmonary embolism (PE), or tubercular infection/reactivation, were detected during this study observation.

## 4. Discussion

The present study highlights the efficacy and safety profile of UPA for RA patients in a real-life context. JAK inhibitors are currently approved for the treatment of RA with moderate-to-high disease activity that is inadequately responding to anti-TNF agents. Despite the excellent results in randomized controlled trials [13,14,15,16,17,18,19,20,21,22,23,24], evidence regarding JAK inhibition in RA from real-world data are limited.

Our findings are consistent with previous results, both from an ultrasonographic and a clinimetric standpoint [13,14,15,16,17,18,19,20,21,22,23,24].

Regarding the few real-world data, a study by Amirdzhanova VN et al. verified the effectiveness of Upadacitinib in treating RA over 3 and 6 months. It involved 63 RA patients with high disease activity and was evaluated using various indices, including DAS28(ESR), DAS28(CRP), SDAI, CDAI, HAQ, EQ-5D, and RAPID-3. Results showed significant improvement: by 3 months, 69.8% achieved remission, and by 6 months, remission increased to 90%. Functional improvement was noted in over 70% of patients. This study also observed a reduction in NSAID use and glucocorticoid dosage, highlighting UPAs potential to improve RA patient outcomes in real-world settings [32]. Moreover, the ‘JAK-pot’ study compared the effectiveness of four RA treatments with different mechanisms, including JAKi, in a large international register. Similar retention rates were observed among treatment groups. JAKi (including UPA) and IL-6i were less often discontinued for ineffectiveness than TNFi. Over half of RA patients achieved low disease activity at 1 year [33].

In our study specifically, DAS28-CRP and SDAI significantly improved from the first month of therapy and remained stable throughout the follow-up period, confirming the rapid and sustained efficacy of Upadacitinib in these domains. Similarly, physical function and the severity and duration of morning stiffness improved significantly from baseline to the last follow-up assessment. More specifically, data including HAQ, VAS pain, PtGA, PhGA, and morning stiffness were considerably reduced at the 6-month evaluation.

With regard to ultrasound parameters, we observed a significant decrease in synovial hypertrophy (SH) and power doppler (PD). In particular, differences were significant between T1 and T3 for both parameters, as well as between T0 and T6 for SH and between T1 and T6 for PD. Furthermore, ultrasound and clinimetric data do not correlate with each other, in agreement with previous evidence. Indeed, D’Agostino et al. found no correlations between changes from baseline in DAS28, in the Global OMERACT–EULAR Synovitis Score or component scores for any joint set, or using just the 28 joints used for DAS28 [34]. The efficacy of clinimetric scores was observed more rapidly compared to ultrasonographic changes, which is to be expected given the slower resolution of synovitis and power doppler signaling. This is supported by previous evidence showing a significant improvement in PD scores not before 6 months of follow-up [35].

As for factors predicting response to therapy, patients treated with Upadacitinib in monotherapy displayed a significant improvement in synovial hypertrophy and DAS28-CRP. However, combination therapy with csDMARDs may suggest a more severe and aggressive disease, making it more resistant to therapy and achieving low disease activity or remission. The presence of rheumatoid factor and anti-citrullinated protein antibodies did not impact Upadacitinib efficacy, suggesting excellent efficacy regardless of the serological status.

We did not observe a significant steroid-sparing effect during this study period, which was explained by the already low prednisone doses at baseline.

Finally, UPA exhibited a good safety profile with no remarkable events. Altogether, our data support the favorable benefit-to-risk ratio as previously disclosed by other colleagues [36].

In 2022, the Oral Rheumatoid Arthritis Trial (ORAL surveillance study) found a higher risk of major adverse cardiovascular events and venous thromboembolic events in patients with RA and cardiovascular risk factors treated with Tofacitinib than with TNF inhibitors [37]. Based on these data, the European Medicines Agency and the Pharmacovigilance Risk Assessment Committee have recently endorsed measures to minimize the risk of JAKi-related serious side effects [38]. As a consequence, the 2022 updated version of EULAR recommendations on RA suggests that an age over 65 years, a history of current or past smoking, and the presence of risk factors for cardiovascular events, malignancy, and thromboembolic events must be taken into account when considering treatment with a JAK inhibitor [26].

Interestingly, the safety profile could be different among JAK-inhibitors. This may depend on the different selectivity of the single molecules with different mechanisms, including the NK cell-mediated response, or on a different induction of thrombopoietin [39].

Study limitations include the relatively small sample size and the lack of a control group for comparative analysis. Furthermore, some data relating to the patients’ past therapies is missing, such as the duration of therapy and the reason for discontinuation.

## 5. Conclusions

In conclusion, our findings confirm the good efficacy of Upadacitinib in a real-life context, with a rapid and sustained improvement of clinical and ultrasound parameters, particularly in monotherapy, while showing a good safety profile. Patient-reported outcomes were also significantly impacted. Even if further studies are needed to clarify those results, these novel findings may provide new insight for the management of UPA treatment in clinical practice.

## Figures and Tables

**Table 1:** Baseline clinical and demographic data of this study cohort.

Age (mean ± SD, years)	58.00 ± 10.50
Age at onset (mean ± SD, years)	45.54 ± 2.18
Disease duration (mean ± SD, years)	7.50 ± 9.67
Females (%)	49 (68.05)
BMI median (IQR)	23.17 (21.81–27.32)
Smoking habits: yes, % (n/tot)	25.35% (18/71)
RF + (%)	47 (66.2)
ACPA + (%)	44 (61.92)
RF and ACPA + (%)	37 (51.31)
HZV infection (%)	2 (2.70)
DAS 28	4.1 (1.7)
SDAI (mean ± SD)	19.1 (12.15)
bDMARDs naive (%)	15 (21.1)
1 previous bDMARDS (%)	10 (14)
2 previous bDMARDs (%)	10 (14)
3 previous bDMARDs (%)	18 (25.3)
4 previous bDMARDs (%)	9 (12.7)
5 previous bDMARDs (%)	9 (12.7)
Previous csDMARDs treatment (%)	40 (56.3)
csDMARDs MTX %	36 (90)
csDMARDs HCQ %	2 (5)
csDMARDs LFN %	2 (5)
Concomitant steroid treatment, *n* (%)	29

List of abbreviations: BMI body mass index; ACPA anti-citrullinated protein antibodies; DAS 28-CRP Disease Activity Score 28—C-reactive protein; HZV herpes zoster virus; RF rheumatoid factor; SD standard deviation; SDAI Simplified Clinical Activity Index; bDMARDs biological disease-modifying anti-rheumatic drugs; csDMARDs conventional synthetic disease-modifying antirheumatic drugs; MTX methotrexate; HCQ hydroxychloroquine; LFN leflunomide.

**Table 2:** Number of patients continuing and discontinuing treatment with UPA according to sex, antibody profile, and HZV infection.

Variable		Ongoing Treatment with UPA	UPA Discontinuation
Sex	Female (%)	44 (68.75)	6 (85.7)
	Male (%)	20 (31.25)	1 (14.3)
RF	Positive (%)	47 (66.2)	0
	Negative (%)	20 (28.1)	4 (5.63)
ACPA	Positive (%)	43 (67.2)	1 (28.6)
	Negative (%)	21 (32.8)	5 (71.4)
HZV infection	Yes (%)	0	0
	No (%)	71 (100)	0
csDMARDs	Yes (%)	37 (52.1)	3 (4.2)
	No (%)	27 (38)	4 (5.63)
Steroid	Yes (%)	25 (35.2)	4 (5.63)
	No (%)	39 (54.9)	3 (4.22)

List of abbreviations: ACPA anti-citrullinated protein antibodies; csDMARDs conventional disease modifying anti-rheumatic drugs; HZV herpes zoster virus; RF rheumatoid factor; UPA Upadacitinib.

**Table 3:** Changes in clinimetric and ultrasonographic scores throughout this study period and significance levels (*p* values) for single comparisons between different observations in time. Differences between groups for repeated variables were investigated by the Friedman test, followed by post-hoc analysis with the Wilcoxon Sign test.

Endpoint	Overall Significance	T0 vs. T1	T0 vs. T3	T0 vs. T6	T1 vs. T3	T1 vs. T6	T3 vs. T6
Synovitis grade	<0.001	0.804	0.090	<0.001	<0.001	<0.001	<0.001
PD signal grading	0.029	0.914	0.320	0.026	0.029	0.009	0.066
Tenosynovitis grading	0.012	0.968	0.370	0.031	0.018	0.009	0.059
DAS28-CRP	0.005	0.014	<0.001	<0.001	0.220	0.015	0.035
SDAI index	<0.001	0.003	<0.001	<0.001	<0.001	<0.001	<0.001

List of abbreviations: DAS28-CRP Disease Activity Score 28—C-reactive protein; PD power Doppler; SDAI Simplified Clinical Activity Index; T0 baseline; T1 1-month evaluation; T3 3-month evaluation; T6 6-month evaluation.

**Table 4:** Linear regression variables are used to predict changes in synovitis grading, Power Doppler grading, DAS28-CRP, and SDAI.

Independent Variable	Predictive Variables	*p* Value	Odds Ratio	Confidence Interval
Δ Synovitis grade	Biologic line	0.880	−0.352	[−4.997–4.292]
	Concomitant csDMARDs at baseline	0.010	−4.824	[−8.427–−1.221]
	Comorbidities	0.681	−0.698	[−4.083–2.687]
	Elevated ESR and/or CRP	0.699	0.745	[−3.090–4.579]
Δ PD grade	Biologic line	0.112	−1.073	[−2.404–0.257]
	Concomitant csDMARDs at baseline	0.057	−1.003	[−2.035–0.029]
	Comorbidities	0.151	−0.704	[−1.674–0.265]
	Elevated ESR and/or CRP	0.972	0.019	[−1.079–1.118]
Δ DAS28-CRP	Biologic line	0.328	−0.434	[−1.314–0.446]
	Concomitant csDMARDs at baseline	0.045	−0.680	[−1.346–−0.014]
	Comorbidities	0.343	−0.297	[−0.920–0.326]
	Elevated ESR and/or CRP	0.252	0.410	[−0.300–1.121]
Δ SDAI	Biologic line	0.473	−2.474	[−9.342–4.393]
	Concomitant csDMARDs at baseline	0.050	−2.712	[−10.939–0.004]
	Comorbidities	0.269	−5.195	[−7.574–2.149]
	Elevated inflammatory markers	0.003	8.481	[2.940–14.022]

List of abbreviations: csDMARDs conventional disease-modifying anti-rheumatic drugs; CRP C-reactive protein; DAS28-CRP disease activity score28-CRP; ESR erythrocyte sedimentation rate; PD Power Doppler; SDAI simplified clinical disease activity index.

## Data Availability

All the data are present in the main text.
